# Supplementary material for: IgE actions on CD4+ T cells, mast cells, and macrophages participate in the pathogenesis of experimental abdominal aortic aneurysms
Source: EMBO Mol Med. 2014 Jun 24;6(7):952–69. doi: 10.15252/emmm.201303811 (PMC4119357; doi:10.15252/emmm.201303811)
Supplement: Supplementary file 1 — Supplementary Figure S1 [file emmm0006-0952-SD1.pdf]

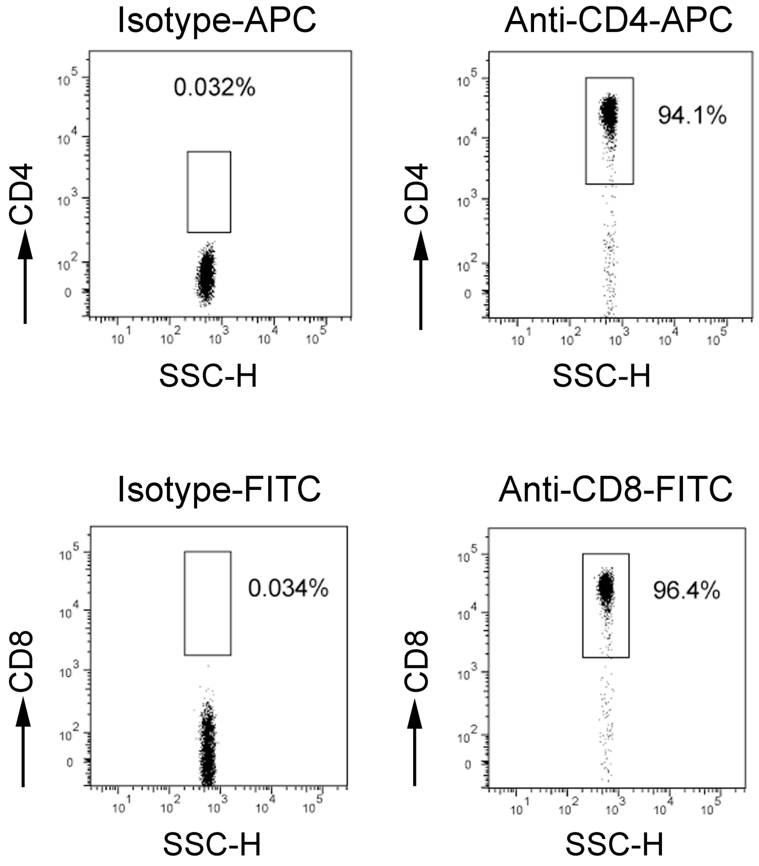

Fig. S1. FACS analysis using anti-CD4-APC mAb and anti-CD8-FITC mAb to assess the purity of $CD4^+$ and $CD8^+$ T cells purified from mouse splenocytes (right panels). Corresponding antibody isotypes (left panels) were used as experimental controls.
