# Supplementary material for: IgE actions on CD4+ T cells, mast cells, and macrophages participate in the pathogenesis of experimental abdominal aortic aneurysms
Source: EMBO Mol Med. 2014 Jun 24;6(7):952–69. doi: 10.15252/emmm.201303811 (PMC4119357; doi:10.15252/emmm.201303811)
Supplement: Supplementary file 2 — Supplementary Figure S2 [file emmm0006-0952-SD2.pdf]

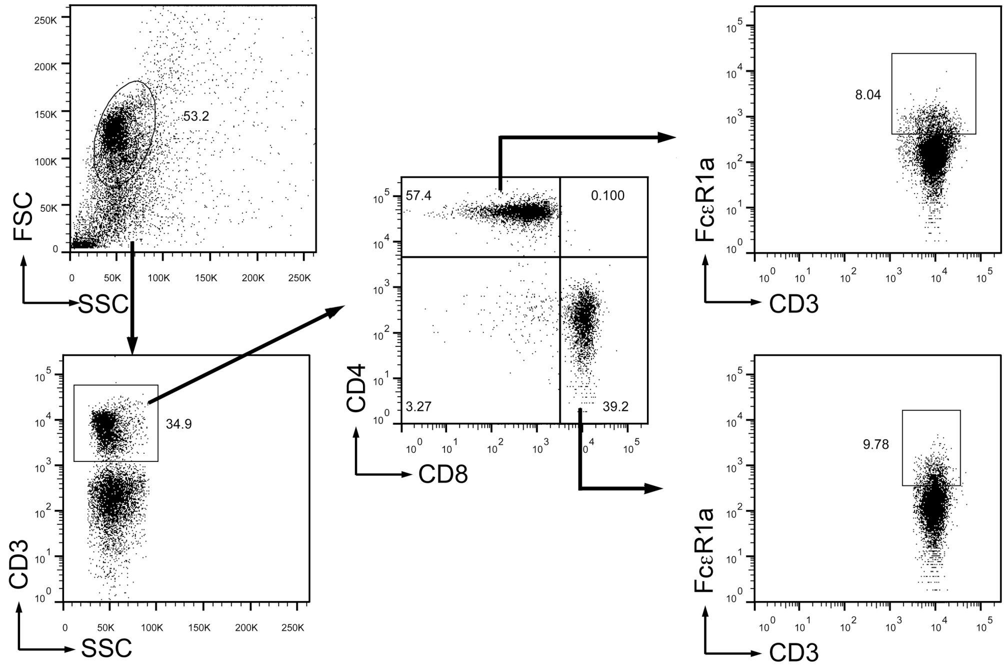

Fig. S2. Schemes of $CD4^+$ and $CD8^+$ T-cell gating and FACS analysis of FcεR1a-positive $CD4^+$ and $CD8^+$ T cells from total splenocytes.
