# Supplementary material for: IgE actions on CD4+ T cells, mast cells, and macrophages participate in the pathogenesis of experimental abdominal aortic aneurysms
Source: EMBO Mol Med. 2014 Jun 24;6(7):952–69. doi: 10.15252/emmm.201303811 (PMC4119357; doi:10.15252/emmm.201303811)
Supplement: Supplementary file 3 — Supplementary Figure S3 [file emmm0006-0952-SD3.pdf]

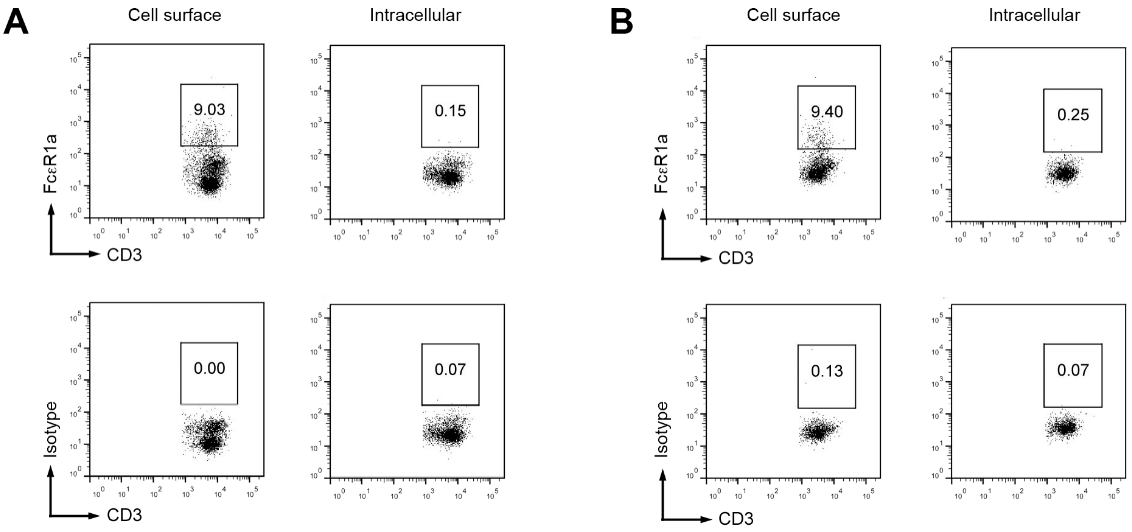

Fig. S3. Representative FACS analysis of cell surface (left panels) and intracellular (right panels) expression of FcεR1a from $CD3^{+}CD4^{+}$ T cells (**A**) and $CD3^{+}CD8^{+}$ T cells (**B**). Antibody isotype controls are shown to the bottom panels.
