# Supplementary material for: IgE actions on CD4+ T cells, mast cells, and macrophages participate in the pathogenesis of experimental abdominal aortic aneurysms
Source: EMBO Mol Med. 2014 Jun 24;6(7):952–69. doi: 10.15252/emmm.201303811 (PMC4119357; doi:10.15252/emmm.201303811)
Supplement: Supplementary file 4 — Supplementary Figure S4 [file emmm0006-0952-SD4.pdf]

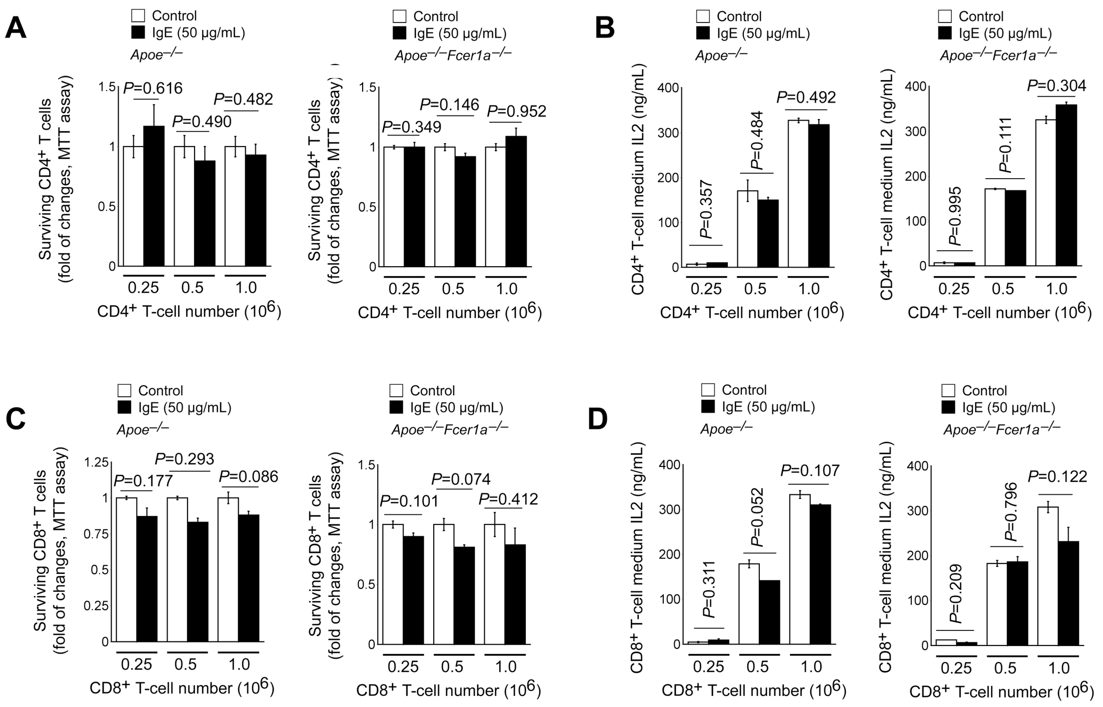

Fig. S4. IgE showed no effect on $CD4^+$ and $CD8^+$ T-cell survival or proliferation. Cells were incubated in 200 µl RPMI-1640 with anti-mouse CD3 (1 µg/mL) and anti-mouse CD28 (1 µg/mL) antibodies on a 96-well plate and treated with or without mouse IgE (50 µg/mL) for 3 days. **A**. MTT assay to assess survival changes of different amounts of $CD4^+$ T cells (indicated) treated with and without IgE (50 µg/mL). **B**. ELISA determined $CD4^+$ T-cell medium IL2 levels after cells were treated with different amounts of IgE, as indicated. **C**. MTT assay to assess survival changes of different amounts of $CD8^+$ T cells (indicated) treated with and without IgE. **D**. ELISA determined $CD8^+$ T-cell medium IL2 levels after cells were treated with different amounts of IgE, as indicated. Data are mean ± SEM of three independent experiments.
