# Supplementary material for: IgE actions on CD4+ T cells, mast cells, and macrophages participate in the pathogenesis of experimental abdominal aortic aneurysms
Source: EMBO Mol Med. 2014 Jun 24;6(7):952–69. doi: 10.15252/emmm.201303811 (PMC4119357; doi:10.15252/emmm.201303811)
Supplement: Supplementary file 5 — Supplementary Figure S5 [file emmm0006-0952-SD5.pdf]

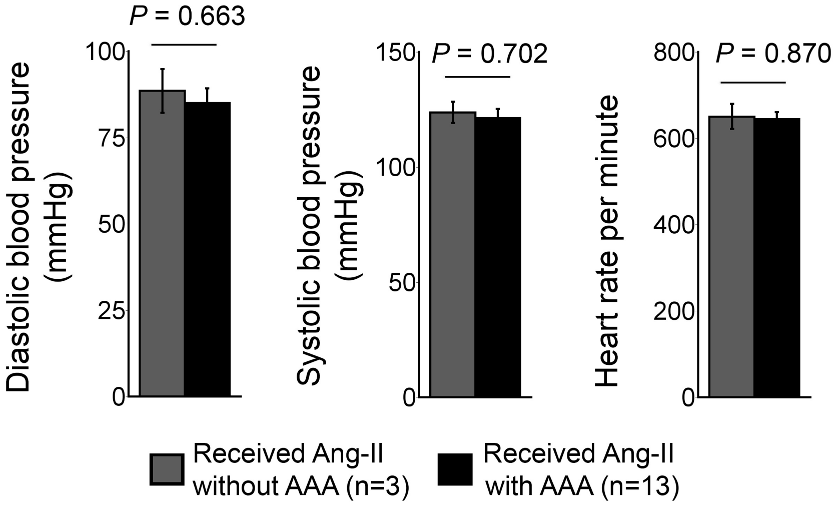

Fig. S5. Diastolic and systolic blood pressures and heart rates in *Apoe*$^{-/-}$ mice that developed AAA (n=13) and did not develop AAA (n=3) after Ang-II infusion.
