# Supplementary material for: IgE actions on CD4+ T cells, mast cells, and macrophages participate in the pathogenesis of experimental abdominal aortic aneurysms
Source: EMBO Mol Med. 2014 Jun 24;6(7):952–69. doi: 10.15252/emmm.201303811 (PMC4119357; doi:10.15252/emmm.201303811)
Supplement: Supplementary file 6 — Supplementary Figure S6 [file emmm0006-0952-SD6.pdf]

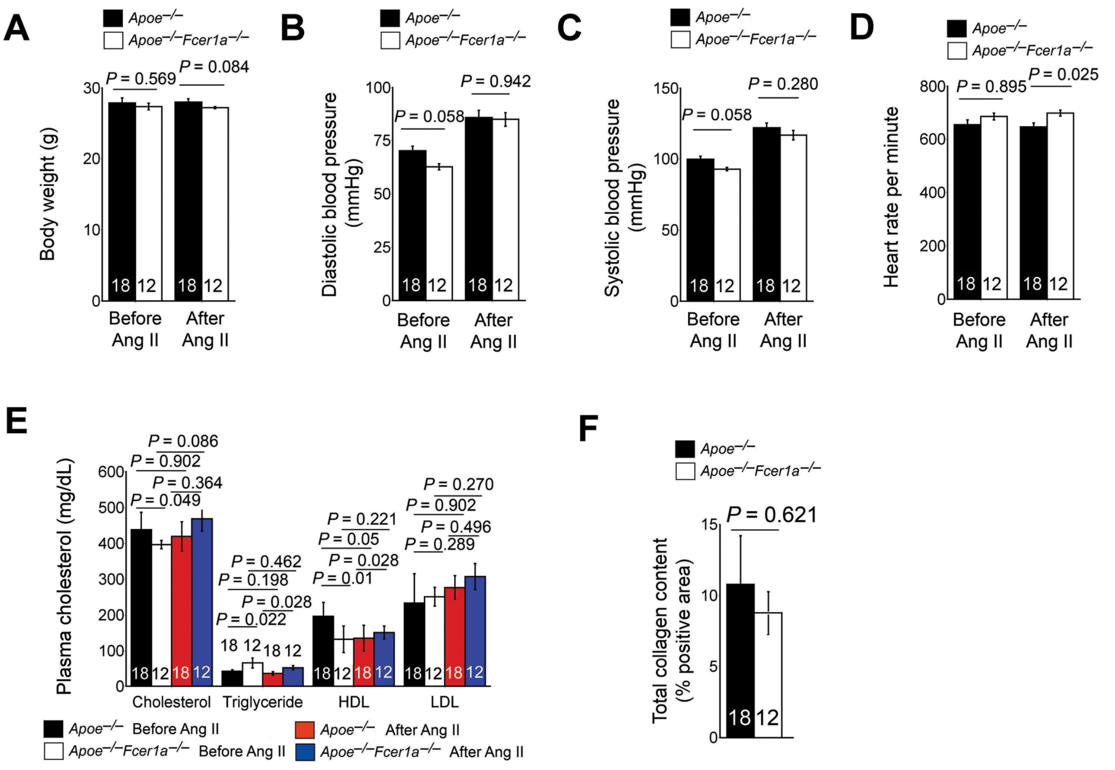

Fig. S6. Body weight (**A**), diastolic blood pressure (**B**), systolic blood pressure (**C**), and heart rate (**D**) of *Apoe*$^{-/-}$ and *Fcer1a*$^{-/-}$*Apoe*$^{-/-}$ mice before and after AAA production (Ang-II infusion). **E**. Serum total cholesterol, triglyceride, high-density lipoprotein (HDL), and low-density lipoprotein (LDL) levels in *Apoe*$^{-/-}$ and *Fcer1a*$^{-/-}$*Apoe*$^{-/-}$ mice before and after Ang-II infusion and AAA production. **F.** Sirius red staining determined total collagen areas in AAA lesions from *Apoe*$^{-/-}$ and *Fcer1a*$^{-/-}$*Apoe*$^{-/-}$ mice. Data are mean ± SEM. The number of mice per group is indicated in each bar.
