# Supplementary material for: IgE actions on CD4+ T cells, mast cells, and macrophages participate in the pathogenesis of experimental abdominal aortic aneurysms
Source: EMBO Mol Med. 2014 Jun 24;6(7):952–69. doi: 10.15252/emmm.201303811 (PMC4119357; doi:10.15252/emmm.201303811)
Supplement: Supplementary file 7 — Supplementary Figure S7 [file emmm0006-0952-SD7.pdf]

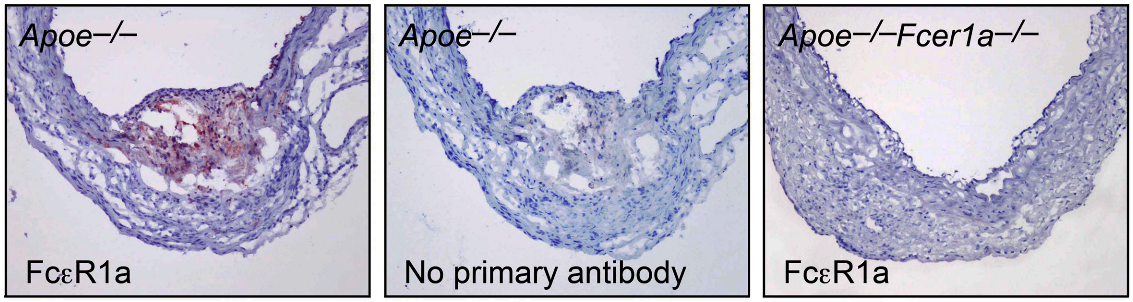

Fig. S7. Hamster anti-mouse FcεR1a antibody specificity test. **Left:** anti-mouse FcεR1a antibody immunostaining of AAA lesions from *Apoe*$^{-/-}$ mice. **Middle:** Consecutive section was immunostained without the primary antibody. **Right:** anti-mouse FcεR1a antibody immunostaining of AAA lesions from *Fcer1a*$^{-/-}$*Apoe*$^{-/-}$ mice.
