# Supplementary material for: IgE actions on CD4+ T cells, mast cells, and macrophages participate in the pathogenesis of experimental abdominal aortic aneurysms
Source: EMBO Mol Med. 2014 Jun 24;6(7):952–69. doi: 10.15252/emmm.201303811 (PMC4119357; doi:10.15252/emmm.201303811)
Supplement: Supplementary file 8 — Supplementary Figure S8 [file emmm0006-0952-SD8.pdf]

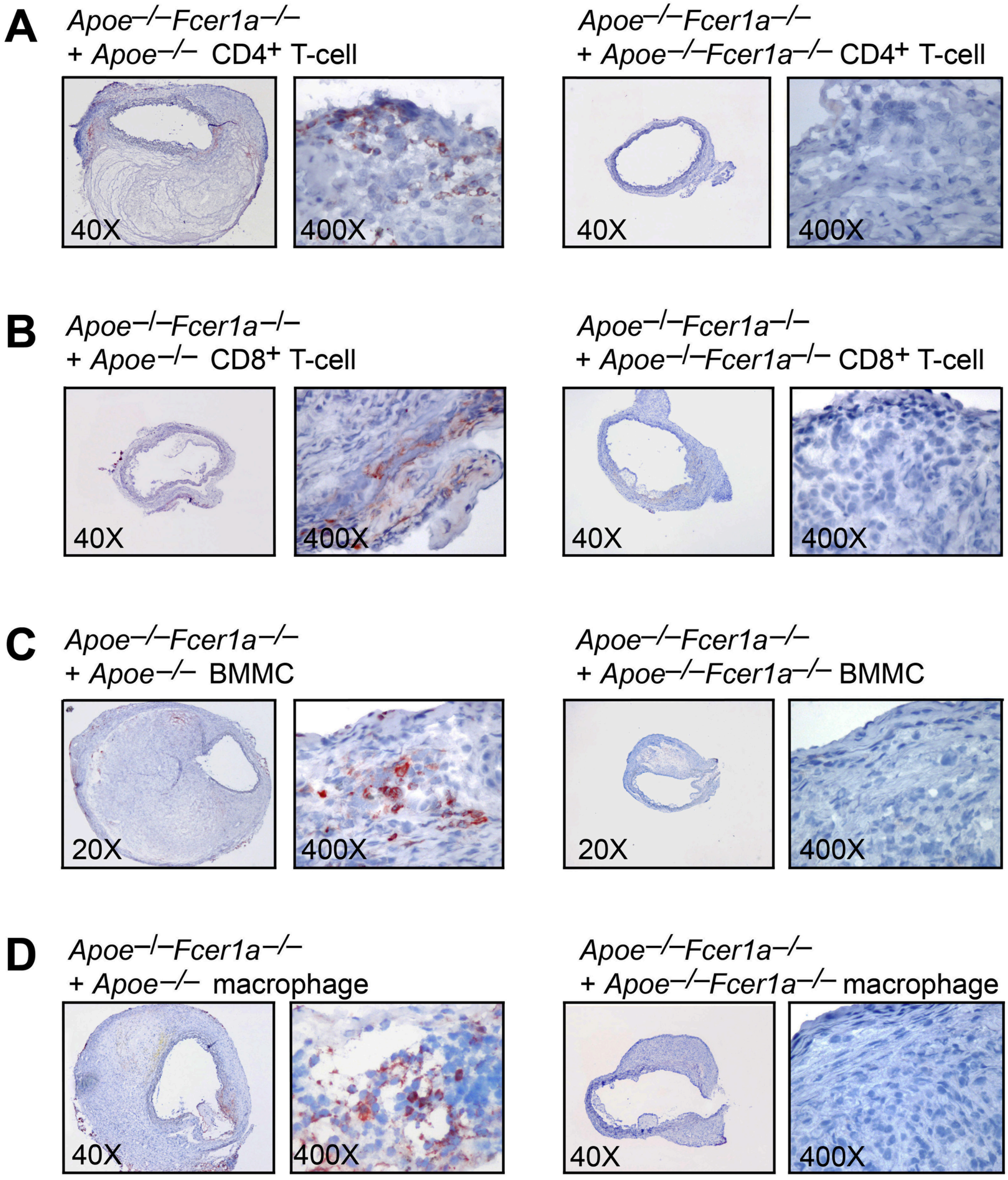

Fig. S8. Hamster anti-mouse FcεR1a antibody immunostaining to detect donor CD4^{+} T cells (**A**), CD8^{+} T cells (**B**), BMMCs (**C**), and macrophages (**D**) from *Apoe*$^{-/-}$ (left panels) and *Fcer1a*$^{-/-}$*Apoe*$^{-/-}$ mice (right panels). Different magnifications were indicated.
