# Supplementary material for: IgE actions on CD4+ T cells, mast cells, and macrophages participate in the pathogenesis of experimental abdominal aortic aneurysms
Source: EMBO Mol Med. 2014 Jun 24;6(7):952–69. doi: 10.15252/emmm.201303811 (PMC4119357; doi:10.15252/emmm.201303811)
Supplement: Supplementary file 9 — Supplementary Figure S9 [file emmm0006-0952-SD9.pdf]

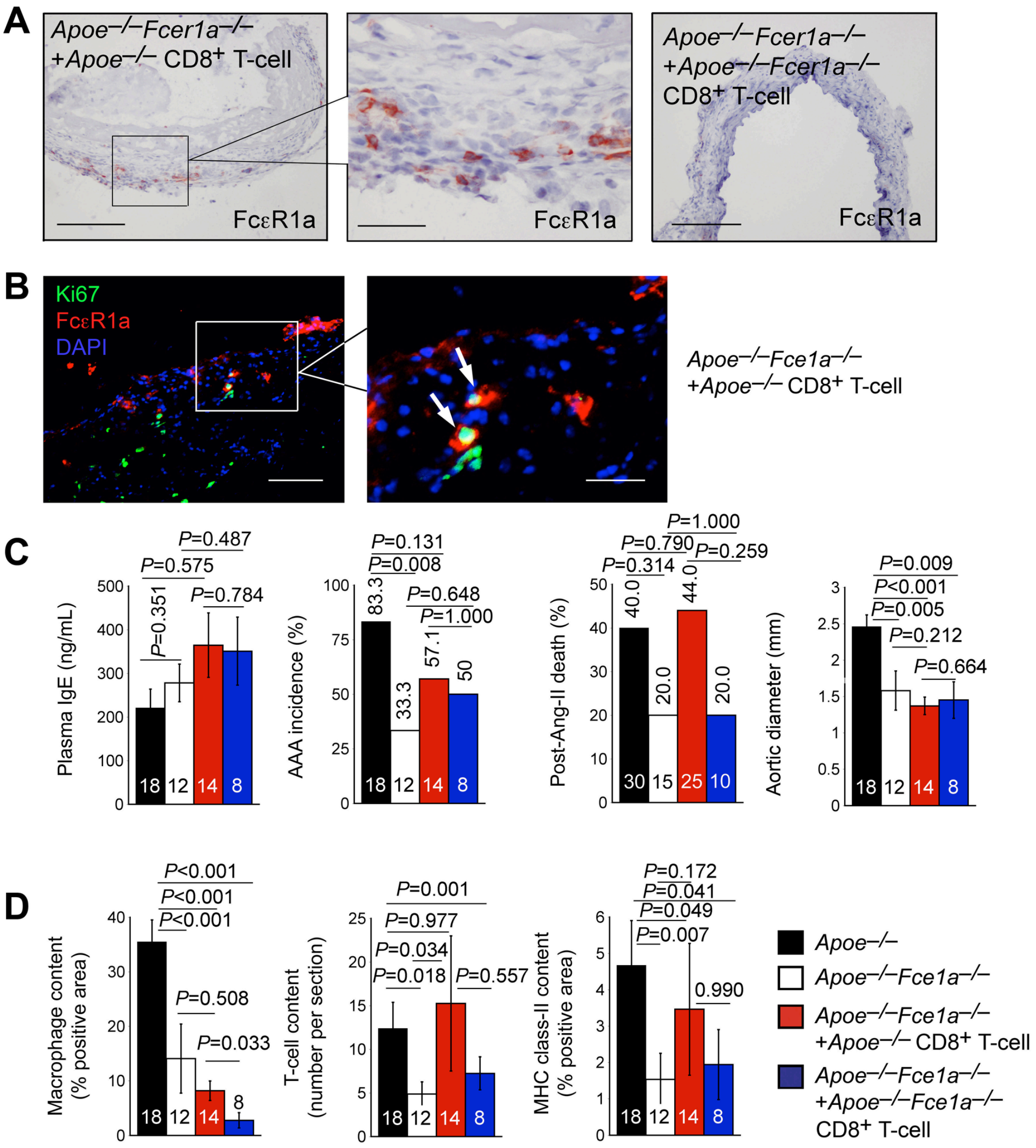


**Fig. S9.** IgE actions on CD8$^+$ T cells in AAAs. **A**. Representative immunostaining with anti-FcεR1a antibody to detect donor CD8$^+$ T cells from *Apoe*$^{-/-}$ (left two panels) and *Fcer1a*$^{-/-}$*Apoe*$^{-/-}$ mice (right panel) in AAA lesions from *Fcer1a*$^{-/-}$*Apoe*$^{-/-}$ recipient mice. Scale: 200 μm; insert scale: 50 μm. **B**. Immunofluorescent double staining to detect FcεR1a$^+$Ki67$^+$ proliferating donor CD8$^+$ T cells in AAA lesions from *Fcer1a*$^{-/-}$*Apoe*$^{-/-}$ recipient mice receiving donor CD8$^+$ T cells from *Apoe*$^{-/-}$ mice. Scale: 200 μm; insert scale: 60 μm. **C**. Plasma IgE levels, AAA incidence, mortality rate after Ang-II infusion, and maximal suprarenal outer aortic diameter measured from in situ; and **D**. AAA lesion macrophage content, CD4$^+$ T-cell content, and MHC class-II-positive area from *Apoe*$^{-/-}$ and *Fcer1a*$^{-/-}$*Apoe*$^{-/-}$ mice and *Fcer1a*$^{-/-}$*Apoe*$^{-/-}$ recipient mice receiving donor CD8$^+$ T cells from *Apoe*$^{-/-}$ and *Fcer1a*$^{-/-}$*Apoe*$^{-/-}$ mice. Data are mean ± SEM. The number of mice per group is indicated in each bar.
