# Supplementary material for: IgE actions on CD4+ T cells, mast cells, and macrophages participate in the pathogenesis of experimental abdominal aortic aneurysms
Source: EMBO Mol Med. 2014 Jun 24;6(7):952–69. doi: 10.15252/emmm.201303811 (PMC4119357; doi:10.15252/emmm.201303811)
Supplement: Supplementary file 10 — Supplementary Figure S10 [file emmm0006-0952-SD10.pdf]

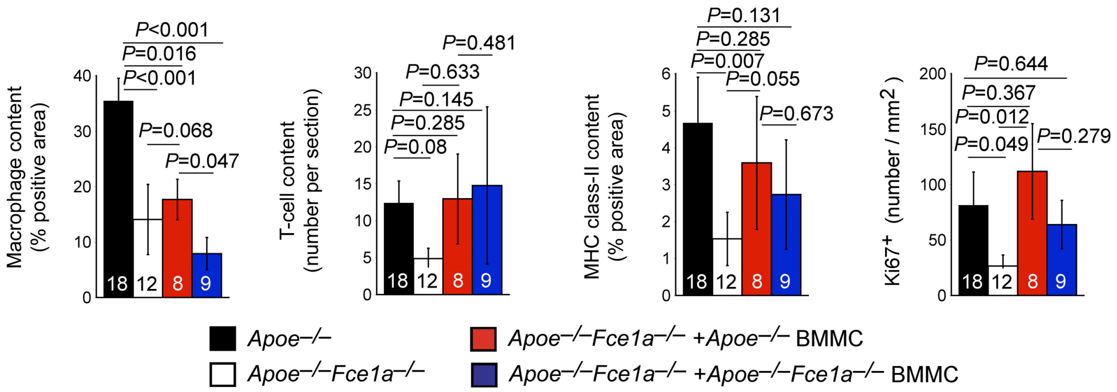

Fig. S10. AAA lesion macrophage content, CD4$^+$ T-cell content, MHC class-II-positive area, and Ki67-positive lesion proliferating cell from *Apoe*$^{-/-}$ and *Fcer1a*$^{-/-}$*Apoe*$^{-/-}$ mice and *Fcer1a*$^{-/-}$*Apoe*$^{-/-}$ recipient mice receiving donor BMMCs from *Apoe*$^{-/-}$ and *Fcer1a*$^{-/-}$ *Apoe*$^{-/-}$ mice. Data are mean ± SEM. The number of mice per group is indicated in each bar.
