# Supplementary material for: IgE actions on CD4+ T cells, mast cells, and macrophages participate in the pathogenesis of experimental abdominal aortic aneurysms
Source: EMBO Mol Med. 2014 Jun 24;6(7):952–69. doi: 10.15252/emmm.201303811 (PMC4119357; doi:10.15252/emmm.201303811)
Supplement: Supplementary file 11 — Supplementary Figure S11 [file emmm0006-0952-SD11.pdf]

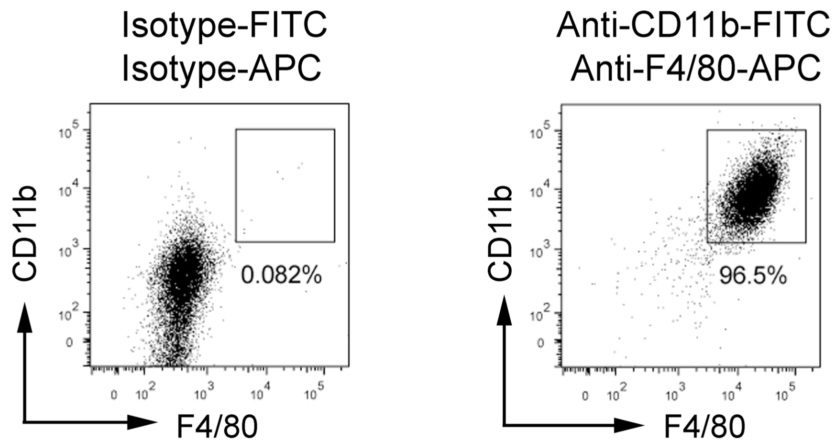

Fig. S11. FACS analysis using anti-CD11b-FITC mAb and anti-F4/80-APC mAb to test the purity of mouse peritoneal macrophages selected by adhesion to deplete non-macrophages. Corresponding antibody isotypes were used as experimental controls.
