# Supplementary material for: IgE actions on CD4+ T cells, mast cells, and macrophages participate in the pathogenesis of experimental abdominal aortic aneurysms
Source: EMBO Mol Med. 2014 Jun 24;6(7):952–69. doi: 10.15252/emmm.201303811 (PMC4119357; doi:10.15252/emmm.201303811)
Supplement: Supplementary file 12 — Supplementary Figure S12 [file emmm0006-0952-SD12.pdf]

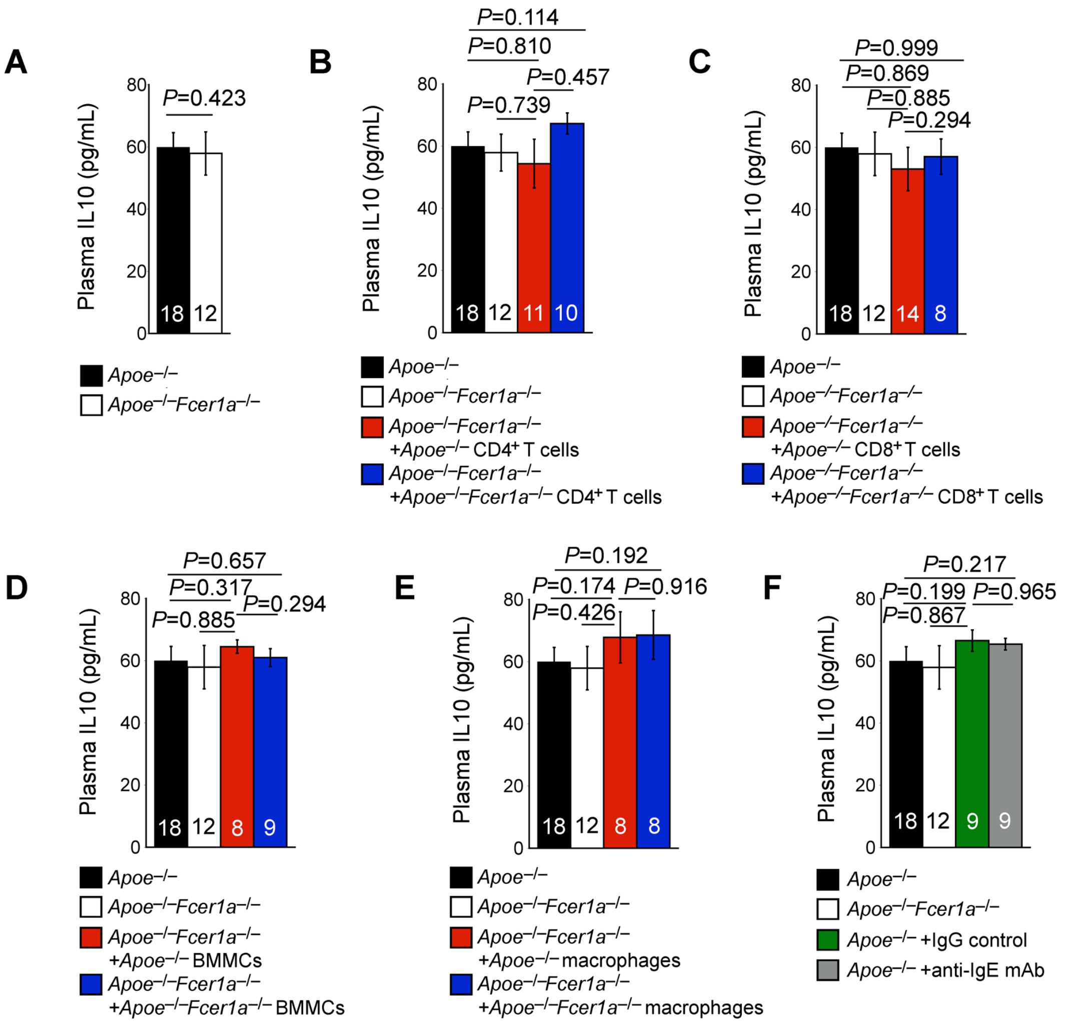

Fig. S12. Plasma IL10 levels in *Apoe*$^{-/-}$ and *Fcer1a*$^{-/-}$*Apoe*$^{-/-}$ mice (**A**), in *Apoe*$^{-/-}$ and *Fcer1a*$^{-/-}$*Apoe*$^{-/-}$ mice and *Fcer1a*$^{-/-}$*Apoe*$^{-/-}$ recipient mice receiving donor CD4^{+} T cells (**B**), CD8^{+} T cells (**C**), BMMCs (**D**), and macrophages (**E**) from *Apoe*$^{-/-}$ and *Fcer1a*$^{-/-}$*Apoe*$^{-/-}$ mice, and in *Apoe*$^{-/-}$ mice that received biweekly intravenous administrations of anti-IgE mAb or corresponding IgG controls (**F**). Data are mean ± SEM. The number of mice per group is indicated in each bar.
