# Supplementary material for: IgE actions on CD4+ T cells, mast cells, and macrophages participate in the pathogenesis of experimental abdominal aortic aneurysms
Source: EMBO Mol Med. 2014 Jun 24;6(7):952–69. doi: 10.15252/emmm.201303811 (PMC4119357; doi:10.15252/emmm.201303811)
Supplement: Supplementary file 13 — Supplementary Figure S13 [file emmm0006-0952-SD13.pdf]

ROC Curve

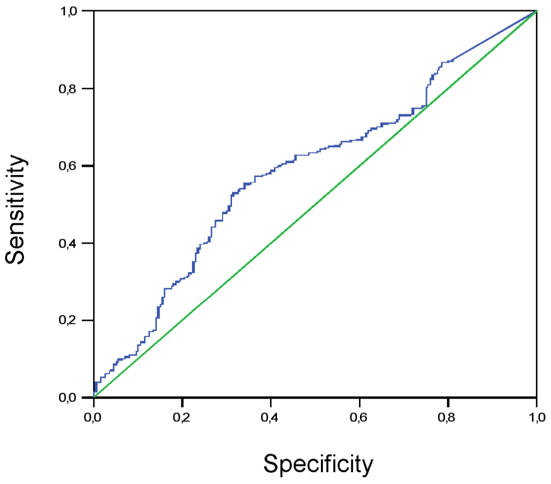

Variable	Area under the curve	Standard error	P-value	95% C.I.	Optimal cut point	Sensitivity	Specificity
IgE (ng/mL)	0.588	0.024	<0.001	0.542 – 0.634	1.60	0.60	0.59

Fig. S13. ROC curve analysis for plasma IgE levels in predicting AAA. AUC (area under the ROC curve) and optimal sensitivity and specificity of IgE are shown in the associated table.
