# Supplementary material for: IgE actions on CD4+ T cells, mast cells, and macrophages participate in the pathogenesis of experimental abdominal aortic aneurysms
Source: EMBO Mol Med. 2014 Jun 24;6(7):952–69. doi: 10.15252/emmm.201303811 (PMC4119357; doi:10.15252/emmm.201303811)
Supplement: Supplementary file 14 — Supplementary Table S1 [file emmm0006-0952-SD14.pdf]

SUPPLEMENTARY TABLE

Table S1. Plasma IgE and log-transformed IgE levels in patients with AAA and PAD.

**IgE**	**AAA**	**N**	**Mean ± SEM**	**Mann-Whitney test *P* value**
[IgE]	No	200	7.098 ± 1.698	
	Yes	487	81.793 ± 61.945	<0.001
Log[IgE]	No	200	1.149 ± 0.079	
	Yes	487	1.557 ± 0.063	<0.001
**IgE**	**PAD**	**N**	**Mean ± SEM**	**Mann-Whitney test *P* value**
[IgE]	No	542	15.321 ± 3.326	
	Yes	131	250.788 ± 229.884	0.007
Log[IgE]	No	542	1.374 ± 0.055	
	Yes	131	1.711 ± 0.130	0.007
